# Estimation of Compressive Strength of Basalt Fiber-Reinforced Kaolin Clay Mixture Using Extreme Learning Machine

**DOI:** 10.3390/ma18020245

**Published:** 2025-01-08

**Authors:** Zeynep Bala Duranay, Yasemin Aslan Topçuoğlu, Zülfü Gürocak

**Affiliations:** 1 Electrical Electronics Engineering Department, Technology Faculty, Firat University, Elazig 23119, Türkiye; 2 Department of Geological Engineering, Firat University, Elazığ 23119, Türkiye; yaslan@firat.edu.tr (Y.A.T.); zgurocak@firat.edu.tr (Z.G.)

**Keywords:** unconfined compressive strength, clay, basalt fiber, extreme learning machine, reinforcement, artificial intelligence

## Abstract

Background: In this study, the unconfined compressive strength (q_u_) of a mixture consisting of clay reinforced with 24 mm-long basalt fiber was estimated using extreme learning machine (ELM). The aim of this study is to estimate the results closest to the data obtained through experimental studies without the need for experimental studies. The literature review reveals that the ELM technique has not been applied to predict the compressive strength of basalt fiber-reinforced clay, and this study aims to provide a novel contribution in this area. Methods: The experimental studies included data derived from a series of mixtures where water contents of 20%, 25%, 30%, and 35% were combined with kaolin clay reinforced with 24 mm-long basalt fiber at reinforcement rates of 0%, 1%, 2%, and 3%. Based on the experimental results obtained for these mixtures, an ELM model was developed to predict the q_u_. Results: ELM, recognized for its computational efficiency and high predictive accuracy, demonstrated exceptional performance in this application, achieving an R value of 0.9976 and an RMSE of 0.0001. Furthermore, this study includes a figure representation illustrating that the ELM-based predictions align closely with the experimental results, underscoring its reliability. Conclusions: To further validate its performance, ELM was compared with other artificial intelligence models through a 5-fold cross-validation approach. The analysis revealed that ELM outperformed its counterparts, achieving a remarkable RMSE value of 0.000174, thereby solidifying its capability to accurately estimate the compressive strength of the soil under varying reinforcement and water content conditions. Thus, it is aimed to save labor, material, and time.

## 1. Introduction

With the increase in the population, the demand for structures and buildings continues to grow, and consequently, the demand for natural construction materials suitable for use in engineering structures is also increasing. However, natural construction material sites with the desired engineering properties are quite limited. As a result, materials obtained from existing sites often require some form of processing. In modern geotechnical engineering, one of the frequently applied practices is embankment construction, where clayey soils with specific engineering properties are required. These requirements are crucial for the safety and cost-effectiveness of embankment projects. Clayey soils that do not possess the necessary engineering properties or partially meet these requirements can be made suitable for use in embankment construction through improvement or reinforcement techniques. The most traditional method of such improvement involves stabilizing clayey soils with low strength, high settlement potential and significant compressibility using various additive materials, such as lime, silica fume, blast furnace slag, volcanic tuff, and fly ash. In this method, pozzolanic reactions occur between the additives and the clayey soil, resulting in aggregation within the soil. This leads to significant improvements in the soil’s strength, swelling, and settlement properties. However, due to increasing costs and environmental concerns, researchers have turned to alternative approaches, leading to a growing interest in more economical, environmentally friendly, sustainable, and readily available materials that are abundant in nature and easy to access.

In recent years, the use of fibers in various fields has become increasingly notable, driven by advancements in technology. While natural fibers have been known to be used in soil reinforcement to a limited extent since ancient times, new technological developments have introduced artificial fibers, such as carbon, glass, and polypropylene. These types of fibers, which can be produced more easily and in quantities sufficient to meet demand, have led to a widespread adoption of fiber usage in soil reinforcement projects as well as in civil engineering projects [1,2]. There has also been a significant increase in research on the use of fibers for soil reinforcement. A review of the literature reveals that polypropylene fiber has been used as a reinforcement material in studies conducted by Tang et al. [3], Diambra et al. [4], Zaimoglu [5], Pradhan et al. [6], Roustaei et al. [7], and Amini and Noorzad [8]. In other studies, nylon fiber [9], polyester fiber [10], carbon fiber [11,12], and polymer and glass fibers [13] have been utilized for soil reinforcement purposes.

Lately, basalt fiber (BF) has emerged as another type of artificial fiber widely used in soil reinforcement due to its numerous advantages. BF is an inorganic fiber obtained from basalt rocks [14]. Basalt, which forms at depths of approximately 100 to 300 km beneath the Earth’s surface, is a dense, solid, naturally occurring rock that can vary in color from black to brown. It can turn the surface into molten lava with a melting temperature ranging from 1500 to 1700 °C [15,16]. Chemically, the primary component of basalt is SiO_2_, followed by Al_2_O_3_ and then Fe_2_O_3_, FeO, CaO, and MgO [17]. While BFs and E-glass fibers show similarities in chemical composition, they exhibit some differences in their primary components. For example, B_2_O_3_, which is typically added as a flux during processing, is absent in BFs [16]. Additionally, BFs exhibit higher tensile strength and better chemical and heat resistance compared to steel fibers [18]. The most commonly used BFs typically have diameters ranging from 13 to 20 µm and lengths ranging from 10 to 65 mm. Basalt’s density ranges from approximately 1.3 to 2.75 g/cm^3^, with tensile strength varying between 2600 and 4840 MPa and an elastic modulus ranging from 80 to 115 GPa [19]. The widespread availability of basalt rock in nature; its abundance; its lack of harm to the environment, humans, and living beings; as well as its sustainability and high strength make BF a significant material. However, studies investigating the changes and improvements in soil properties resulting from the use of BF as a reinforcement material have gained prominence in recent years.

In one of these studies, Gisymol and Ramya [20] reported that a fiber reinforcement of 0.05% and a fiber length of 10 mm resulted in the maximum improvement in the strength of organic soil after a 28-day curing period. Gao et al. [21] stated that a fiber content of 0.25% and a fiber length of 12 mm provide the maximum improvement in clay strength. In the study by Zheng et al. [22], an innovative method was proposed to improve the stability of tailings dams using BF to reinforce the tailings. The experimental results indicated that the mechanical properties of BF-reinforced tailings increased with the increases in fiber length and content, particle size, dry density, and confining pressure. The experimental study by Xu et al. [23] on low-plasticity loess samples reinforced with fibers of three different lengths and four varying contents showed that a fiber length of 12 mm and a content of 0.6% significantly improved shear strength. Gürocak and Aslan Topçuoğlu [24] aimed to determine the changes in soil strength by using BF reinforcement in low-plasticity clay. Experimental studies conducted on samples prepared with varying water and fiber contents revealed that the maximum q_u_ was achieved at 25% water content and 1% BF content. In the study by Aslan Topçuoğlu and Gürocak [25], the effects of BF content on the strength of high-plasticity bentonite clay reinforced with 6 mm BF were investigated. The experimental results showed that maximum strength was achieved with 4% BF reinforcement. In their study, Sun et al. [26] conducted unconfined compressive strength (UCS) and splitting tensile strength (STS) tests on BF-reinforced, cemented silty sand with various fiber contents and curing durations. It was determined that the UCS and STS values of BF-reinforced, uncemented silty sand and BF-reinforced, cemented silty sand increased with the curing time and reached their maximum values after a 28-day curing period.

These studies predominantly focus on the effects of BF on the q_u_ of soils, and the obtained results have been evaluated using traditional methods. However, to establish a sufficient knowledge base and better understand the results and their underlying reasons, it is necessary to evaluate experimental results using innovative methods, such as artificial neural networks (ANN), genetic algorithms, fuzzy logic, and particle swarm optimization. Various models have been used in studies where different types of fibers were used as reinforcement materials. One of these studies is the research conducted by Chou et al. [27]. In their study, a comprehensive fiber-reinforced soil (FRS) database was developed based on high-quality triaxial and direct shear tests documented in the literature between 1983 and 2015. The database includes information on the properties of sand, fibers, the soil–fiber interface, and stress parameters. Their study has been noted to contribute to the development of an effective artificial intelligence (AI) model for predicting the peak friction angle of FRS. Garg et al. [28] developed extreme learning machine (ELM) models to estimate the compressive strength of soil reinforced with various fiber types, including coconut, jute, water hyacinth, and polypropylene. These ELM models were found to provide sufficient accuracy in predicting the compressive strength of fiber-reinforced soil.

Armaghani et al. [29] carried out experimental studies to determine the cohesion values of sandy soil reinforced with polypropylene fibers of different lengths and proportions. Fiber content, fiber length, deviator stress, and pore water pressure were selected as model input parameters, and hybrid ANN-based models, namely genetic algorithm (GA)-ANN and particle swarm optimization (PSO)-ANN, were developed. It was stated that GA-ANN could serve as a novel model for effectively predicting the cohesion of fiber-reinforced sandy soil.

In another study by Tiwari and Satyam [30], the effects of pond ash and polypropylene fiber on soil strength were examined, and the experimental data were analyzed using ANN. The results of the ANN modelling showed excellent predictions of the mechanical properties.

Similarly, another study by [31] investigates the application of machine learning models, particularly ANN, for predicting the mechanical properties of concrete. It underscores the advantages of ANN over traditional methods in providing faster and more precise compressive strength predictions.

In their research, Ndepete et al. [32] focused on evaluating the mechanical properties of silty soils reinforced with BF and determining the optimal fiber proportion and length for improving soil properties. To ensure more reliable results, they utilized machine learning models to analyze soil behavior. Their study revealed that fiber length and cell pressure significantly influenced the predictions of maximum deviator stress.

In their study, Sungur et al. [33] prepared test samples with water contents of 13%, 15%, and 17% along with glass fiber reinforcement levels of 0%, 1%, 1.5%, and 2% to predict the shear strength of glass fiber-reinforced clayey soil using the adaptive neuro-fuzzy inference system (ANFIS). Statistical analysis showed that splitting the data into 80% for training and 20% for testing yielded the most accurate predictions of shear strength with the ANFIS model.

Gul and Mir [34] investigated changes in the strength of glass fiber-reinforced soil. An ANN was used to develop a model to predict the relationship between soil strength and various reinforcement parameters in their study. It was stated that the developed ANN could assist researchers in achieving the optimal fiber reinforcement ratio for a specific type of soil.

Yazıcı et al. [35] developed an ANN model using the results of direct shear tests conducted on BF-reinforced clay soil. It was found that using two neurons with a linear transfer function in the output layer and a tangent sigmoid transfer function in the hidden layer provided the best predictions that matched the measured values.

Alisha et al. [36] emphasize the use of ANNs to model soil properties, aiming to improve the prediction accuracy of engineering characteristics, such as soil moisture content and compressive strength, thereby minimizing the need for extensive experimental testing.

In the study conducted by Houcine et al. [37], a predictive model utilizing ANNs and a simulation-based approach was introduced to estimate the tensile and compressive strengths of eco-friendly concrete containing various natural fibers. The results obtained from the experiments demonstrated that the ANN model outperformed other models and could serve as a reliable approach for predicting the compressive strength of eco-friendly earth concrete.

The study by Kannan and Sujatha [38] aimed to determine the UCS and California bearing ratio of fiber-reinforced soil using machine learning techniques. The findings revealed that the Bayesian regularization algorithm of the ANN technique demonstrated the best performance in predicting the strength of fiber-reinforced soil.

Sert et al. [39] created several mixtures for experimental analysis using varying water contents, different fiber lengths (6, 12, and 24 mm), and proportions (1%, 2%, and 3%) in their study. To predict stress and deformation parameters, five machine learning algorithms were applied: linear regression (LR), ridge regression, lasso regression, support vector regression, and decision tree (DT) regression. The results indicated that the best predictions were obtained using support vector regression and DT regression.

Aslan Topçuoğlu et al. [40] investigated the effect of BF reinforcement on the q_u_ of clay using an ANN model. Their study indicated that there was good agreement between the experimental results and the ANN model predictions.

Arslan et al. [41] used metaheuristic optimization algorithms, such as the equilibrium optimizer, marine predators algorithm, and manta ray foraging optimization, along with a decision tree model to identify optimal reinforcement strategies for fiber-reinforced clays (basalt, polypropylene, and glass fibers) in three different clay types. The results highlighted BF as the most effective reinforcement method, while the findings underscored the effectiveness of metaheuristic algorithms in optimizing predictive models and maintaining accuracy.

Khawaja et al. [42] investigated the effectiveness of genetic programming (GEP), multi-expression programming, and ANN models in predicting the modulus of resilience (MR) of subgrade soils. Their study concluded that GEP is a reliable tool for accurate prediction of MR in subgrade applications.

In the study by Li et al. [43], the back-propagation ANN method was employed to fully consider various influential factors affecting the compressive strength and flexural strength of ultra-high-performance concrete with coarse aggregates. Their study highlighted that the proposed ANN model can be used to determine the optimal mix proportion and predict the strength of ultra-high-performance concrete with coarse aggregates.

Considering the studies summarized above, it is evident that research evaluating the use of BFs in soil reinforcement through AI methods is quite limited. The use of AI not only eliminates the need for materials and equipment required for experimental procedures but also significantly reduces the time spent on such studies, thereby increasing overall efficiency.

In this study, experimental data from the literature on the q_u_ of BF-reinforced clay soils were utilized to estimate the q_u_ value using ELM. In the conducted literature review, no studies were identified that applied the ELM technique to predict the compressive strength of clay reinforced with BF. This study aims to address this gap by exploring the potential of the ELM technique in this context, providing a novel contribution to the existing research in the area of geotechnical engineering.

The primary objective of this research is to enable the prediction of q_u_ values based on varying proportions of water, BF, and clay mixtures without the need for additional experimental investigations. The ability to estimate compressive strength without conducting experimental studies offers both economic and practical benefits in this field. The use of AI not only eliminates the requirement for materials and equipment essential for experimental procedures but also significantly reduces the time invested in such studies, thereby enhancing overall efficiency.

This paper is structured as follows. Section 2 examines relevant experimental studies from the literature to present the data used in the research. Section 3 provides an in-depth review of the ELM technique, explaining the model and how it operates. In Section 4, the ELM model applied in this study is introduced, and the results obtained from the experiments are compared with the predictions made by the ELM technique. The model’s accuracy is evaluated through the R and RMSE values. Finally, a 5-fold cross-validation approach is utilized, comparing ELM with other AI techniques.

## 2. Experimental Studies

**Figure 1:**
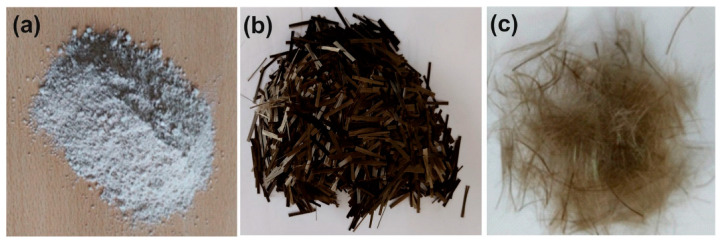
(**a**) Kaolin clay, (**b**) unseparated basalt fiber, and (**c**) separated basalt fibers used in the study.

The experimental studies consist of experiments conducted by Gürocak and Aslan Topçuoğlu [24] to determine the effect of BF addition on the strength of low-plasticity kaolin clay. The studies were carried out in two stages: preparation of both unreinforced and reinforced samples and determination of the q_u_ of these samples. The image of the materials used in the experimental study is shown in Figure 1.

The clay used in the study is as shown in Figure 1a and was produced from the Sındırgı clay quarry in Balıkesir, Turkey.

This clay is of the kaolin type, and the chemical composition was obtained from the company supplying the clay. The composition of the clay is as follows: SiO_2_ 69.00%; Al_2_O_3_ 12–17%; Fe_2_O_3_ 0.50%; CaO 0.10%; Na_2_O 0.20%; K_2_O ≤ 13.17%; trace amounts of TiO_2_, SO_3_ ≤ 7.05%, and Cr_2_O_3_ 0.01%; and trace amounts of BaO. The liquid limit (LL), plastic limit (PL), and plasticity index (PI) values of the clay are 45%, 24%, and 21%, respectively. According to the Unified Soil Classification System, this clay falls into the low-plasticity (CL) clay classification [24]. In the study conducted by Aslan Topçuoğlu and Gürocak [44], the optimum water content (w_opt_) value of this kaolin clay was found to be 25%, and the maximum dry density (γ_kmax_) value was 13.01 kN/m^3^.

BF, with a length of 24 mm, made from basalt, a dark-colored, finely crystalline volcanic rock, as shown in Figure 1b, was used. The BF fibers used in the study were sourced from Spinteks Textile Construction Industry and Trade Inc., Denizli, Türkiye, and the mechanical and physical properties of the fibers were obtained from this company. The characteristics of the fibers are presented in Table 1.

In the sample preparation stage, the BF, as presented in Figure 1c, was separated using a compressor and then dried in an oven at 105 °C for 24 h. The dried BF was added to kaolin clay in proportions of 0%, 1%, 2%, and 3% and mixed. Distilled water was then added to these mixtures in proportions of 20%, 25%, 30%, and 35% using a spraying method. To ensure homogeneous mixing of the fiber, clay, and water, the mixture was blended manually with a mixer for 10 min. Proctor tests were performed on each mixture to compact the mixtures. In the final stage, samples were taken from both the unreinforced and BF-reinforced compacted specimens for unconfined compressive tests. These tests were conducted in accordance with ASTM D2166M-16 [45], and the q_u_ values of the unreinforced and reinforced samples were determined. Thus, the effects of different water contents and BF proportions on the q_u_ of kaolin clay were revealed. The results of the experimental studies are presented in Table 2.

Changes in the q_u_ values of the BF-reinforced samples were observed, depending on the BF proportion and water content. The q_u_ values of the BF-reinforced samples with 20% water content showed a significant increase compared to the unreinforced sample with the same water content. In the sample with 1% BF, the q_u_ value was 523.83 kPa, whereas, when the BF proportion was 2%, the q_u_ value increased to 685.31 kPa. However, when the BF proportion reached 3%, the q_u_ value decreased to 445.06 kPa. In the samples with 25% water content, the q_u_ value was highest (807.40 kPa) when the BF proportion was 1%, showing a significant increase compared to the unreinforced sample. The q_u_ value, which peaked at 1% BF, began to decrease with an increasing fiber proportion. In the samples prepared with 30% water content, the q_u_ values of the BF-reinforced samples increased compared to the unreinforced clay. The q_u_ value reached its maximum (630.23 kPa) in the sample with 2% BF and began to decrease with an increasing fiber proportion. When examining the changes in the q_u_ values of samples with 35% water content, no significant increase was observed with the increasing fiber proportion. Similarly, the q_u_ value of the sample with 2% BF reached its maximum. However, this increase in q_u_ was relatively limited compared to the q_u_ values of the samples with other water contents. In the sample with 3% BF, the q_u_ value was 47.95 kPa, which is almost the same as the q_u_ value of the unreinforced clay. These results are important, as they demonstrate that both the BF proportion and water content have a significant impact on the q_u_ of the soil efficiency.

## 3. Extreme Learning Machine

The data used in engineering applications are usually multidimensional and large-scale, so it is necessary to use complex analysis methods. AI is a technology that is gaining importance in the field of engineering, especially in cases that require long periods of time or complex calculations with traditional analysis methods, as it offers great speed and accuracy in solving complex problems [46].

AI extracts meaningful patterns from complex data structures and makes high-accuracy predictions based on these patterns. In other words, by using AI, engineers in the fields of electrical and electronics engineering, civil engineering, geotechnical engineering, soil engineering, etc. are helped to produce faster, more reliable, and economical solutions [40,47,48].

The q_u_ of a clay–water mixture reinforced with BF can be determined by testing in a laboratory environment; however, this process is quite time-consuming and costly. In addition, complex relationships occur between the strength results and the changes in parameters such as the amount of water used and fiber ratio. AI is effective in dealing with such multivariate data because it learns the relationships between different parameters by analyzing the data. This allows for the rapid estimation of properties that are difficult to measure, such as q_u_, instead of laboratory tests, thus saving time and money for engineering applications.

There are many AI techniques. Among these, ANN, support vector machines (SVM), DT, and LR stand out [40]. Each technique offers its own advantages for certain types of problems.

Among the AI techniques, ELM stands out with its capacity to make fast and accurate predictions [48].

ELM is based on a single-layer neural network structure and completes the training process very quickly due to the random assignment of its weights and the fact that it technically requires less calculations. This speed advantage provides a great advantage in line with the need for fast modeling and prediction in engineering projects.

ELM is a technique based on a single-layer, feed-forward neural network with randomly assigned weights, first proposed by Huang et al. [49]. Unlike traditional neural networks, the ELM technique is popular in applications such as classification, regression, and prediction, as it learns the entire dataset with high accuracy and completes the process faster.

**Figure 2:**
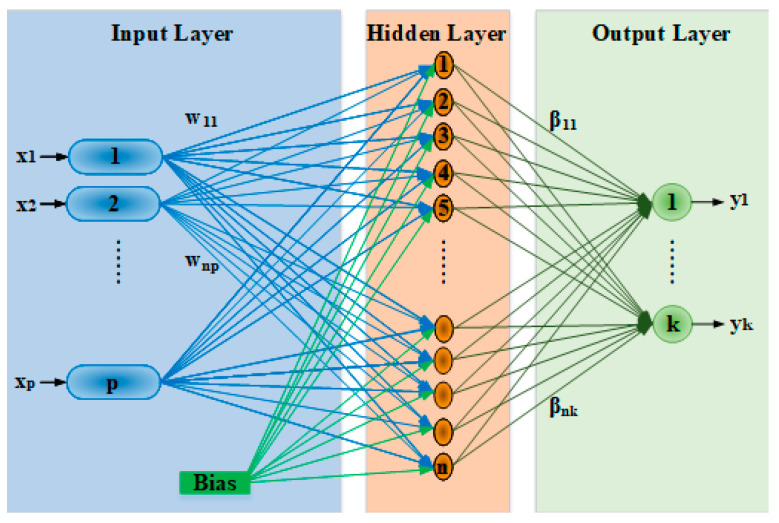
Fundamental structure of an ELM system.

ELM has a feed-forward neural network structure as shown in Figure 2 and consists of three main parts: input layer, hidden layer, and output layer. The fundamental structure of ELM can be described as follows: The training data enter the network through the input layer, which marks the starting point of the model’s learning process. Next, the hidden layer is formed by assigning weights randomly, a characteristic feature that distinguishes ELM from traditional neural networks. These randomly assigned weights facilitate the transformation of data during the learning process. The transformed data are then passed to the output layer, where it is processed to produce the final results of the model.

ELM has a simple training process. First, the weights and bias values from the input layer to the hidden layer are randomly assigned. This random assignment process is the most critical factor that speeds up the training process of ELM. Using the randomly assigned weights and bias values, the outputs of the hidden layer are calculated. This calculation is performed using activation functions such as sigmoid, ReLU, or tanh.

The relationship between the input layer and hidden layer of the ELM for n hidden nodes and *N* inputs is provided in Equation (1) [48]. In the equation, *f* is the activation function, *x* is the input vector, *w* is the weight matrix between the input and the hidden layer, *b* is the bias term in the hidden layer, and *H* is the hidden layer output matrix:

(1) $$H=\sum_{i=1}^{n}f\left(w_{i}.x_{j}+b_{i}\right),j=1,\ldots ,N$$

Then, *H* is multiplied by the weights in the output layer to calculate the final output as given in Equation (2), where *β* is the vector of output weights and *Y* represents the output vector:

(2) Y=Hβ

After the hidden layer outputs are obtained, the output weights are calculated using Equation (3). Equation (3) expresses the pseudo-inverse of the hidden layer output matrix. Here, *H*^†^ is the Moore–Penrose inverse matrix of *H*. This process can also be solved with any linear algebra methods.

(3) $$\beta =H^{†}Y$$

As can be seen, ELM has a fast training process because it assigns weights randomly and establishes a linear relationship with the hidden layer outputs. With these features, it achieves results with high accuracy and fast computation, even from complex datasets.

With these features, ELM is an ideal option for fast and reliable prediction of an engineering parameter such as q_u_.

## 4. ELM-Based Estimation of Compressive Strength

In this study, the q_u_ of clay mixtures reinforced with BF was estimated using an ELM-based approach. For this purpose, the input parameters for the ELM model included the proportions of water, clay, and BF in the mixture experimentally obtained from the literature. Based on these input parameters, the model predicted the q_u_ of the mixture as the output.

**Figure 3:**
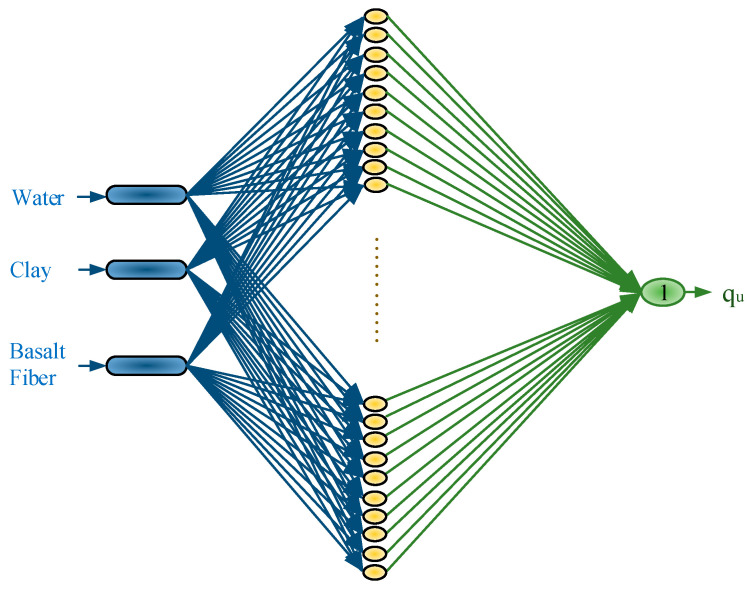
ELM model of this study.

The ELM model utilized in this study is presented in Figure 3. The model comprises three inputs, one hidden layer, and one output. The input variables consist of the proportions of water, clay, and BF. The output of the ELM model predicts the compressive strength of the mixture, providing the q_u_ value.

In this study, the metaheuristic method was used to increase the dataset, which contains a total of 64 experimental data points, to overcome the limitation of the dataset. Thus, the number of data points was expanded to 112. For the expansion, care was taken to preserve the variability and complexity of the original data. During this process, only the training data were increased, and the test dataset was not changed. Thus, the generalization ability of the model was evaluated on the original test data.

Therefore, in this study, a total of 112 data points were utilized, with 70% of the data used for training and the remaining 30% for testing purposes. The activation function, number of hidden neurons, and regularization coefficient used in the model were determined through a trial-and-error approach. Through this process, different combinations of hidden neurons and regularization coefficients were tested, and the optimal configuration was found to be 10,000 hidden neurons and a regularization coefficient of 1000. The chosen activation function was the hard limit function, which provided the best results based on the model’s performance.

The performance of the model used in this study was determined using the R and RMSE values. In the evaluation of the AI results, an R value close to ‘1’ and an RMSE value close to ‘0’ indicate superior model performance.

According to the obtained results, the R value of the ELM model was 0.9976, and the RMSE value was 0.0001. When these values are examined, it is evident that the predictions made by the model are close to the actual values.

**Figure 4:**
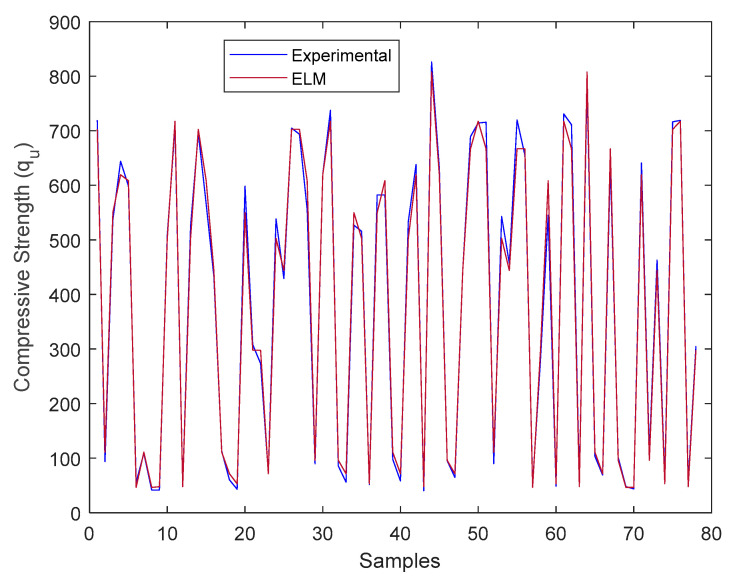
Comparison of the experimental results by the literature with the estimated results by ELM.

The comparison between the predicted compressive strength value from the ELM model and the actual compressive strength value from the literature is presented in Figure 4. Upon examining Figure 4, it can be observed that the ELM predictions closely follow the actual values.

**Figure 5:**
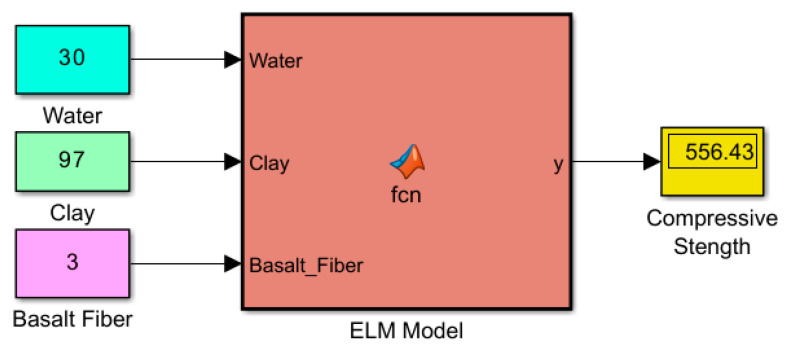
Simulation model of the ELM.

The Matlab simulation model of the compressive strength estimates using ELM, based on water, BF, and clay mixture ratios, is shown in Figure 5, which is an instant that yields the compressive strength for the given input values.

The comparison of the compressive strength prediction values obtained by the ELM model with the experimental results from the literature [24], provided in Table 2, is presented in Table 3. Upon examining both Figure 5 and Table 3, it is observed that the predicted values are almost the same as the actual values. The differences between the ELM model predictions and the experimental results are generally very small, with errors usually within a few decimal places (e.g., 0.01 to 0.5 units). For instance, the predicted value for the experimental output of 102.40 was 102.54, which is a minimal difference of only 0.14 units. This level of accuracy indicates that the ELM model is performing well in terms of predicting the q_u_ of the BF-reinforced clay mixtures.

Moreover, the ELM model has shown consistent stability under varying conditions, such as changes in water content, clay ratio, and BF reinforcement. As seen in Table 3, even with alterations in these factors, the predicted values remain very close to the experimental values, suggesting that the model is robust and performs reliably across different scenarios. This indicates that the ELM model is not only accurate but also stable and resilient to variations in input parameters, making it a reliable tool for predicting the compressive strength of reinforced mixtures under diverse conditions.

In addition, the input data used in this study were carefully curated to ensure that they were reliable and free from inconsistencies. Several preprocessing steps, such as removing outliers, normalizing the data, and addressing missing values, were employed to guarantee that the dataset used for training the ELM model was both accurate and consistent.

Finally, a 5-fold cross-validation approach was employed to evaluate the robustness of the model. Cross-validation is a critical technique in model evaluation as it helps prevent overfitting, ensures the model’s robustness, and assesses its ability to generalize to unseen data [50]. The cross-validation process involved splitting the dataset into five subsets, training the model on four subsets, and testing on the remaining subset. This procedure is repeated five times to ensure reliable and consistent evaluation, which is why it is referred to as the 5-fold cross-validation method.

To demonstrate the performance and reliability of the model employed in this study, various AI methods, including ANN, Gaussian process regression (GPR), ensemble, SVM, LR, and DT, were compared with ELM using a 5-fold cross-validation approach.

**Figure 6:**
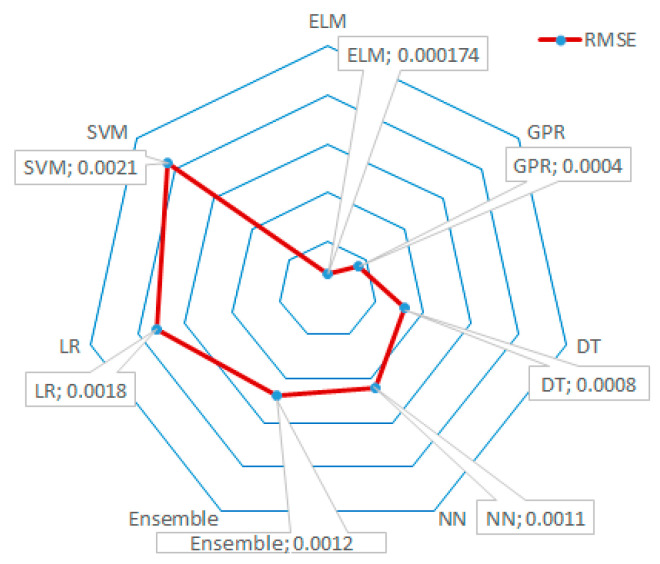
Radar chart of RMSE values of various AI methods.

The evaluation of the obtained data from the comparison analysis of 5-fold cross-validation was realized by the RMSE values, which serve as a key metric for measuring prediction accuracy. The RMSE values for each method are provided in Figure 6. According to the results, the SVM method exhibited the highest RMSE value, indicating the poorest performance, while ELM, with an RMSE value of 0.000174, produced results closest to zero, thereby underscoring its superior predictive capability and accuracy.

## 5. Conclusions

In this study, an ELM model was developed to predict the q_u_ of low-plasticity kaolin clay reinforced with BF. The input data for the ELM model included the fiber ratio, kaolin clay ratio, and water content, while the output data consisted of the q_u_ value. The q_u_ values obtained from the experimental study were evaluated using the ELM. The developed ELM model achieved an R of 0.9976 and an RMSE of 0.0001, demonstrating its accuracy in predicting compressive strength.

- The q_u_ values predicted by the ELM showed a high degree of agreement with the experimental results, highlighting the effectiveness of ELM in assessing compressive strength performance.
- A 5-fold cross-validation technique was applied, and the RMSE value for ELM was calculated as 0.000174, further validating the robustness of the ELM model compared to other AI techniques.
- The ELM technique used in this study has not been previously applied for predicting the compressive strength of BF-reinforced clay in the literature, making this approach novel in this context.
- The ELM model demonstrated excellent stability across various conditions (e.g., changes in BF ratio and water content), indicating its reliability in predicting q_u_ values under different scenarios.

The ELM model used in this study enables the rapid prediction of q_u_ values at different curing times and known mixture ratios without the need for prior experiments. In this regard, the adoption of ELM offers significant advantages in terms of time savings, cost reduction, and labor efficiency. As a result, this study aims to provide a practical tool for engineers and researchers, contributing to more informed decisions in geotechnical engineering and optimizing resource use. By varying both the BF ratio and water content, the results from studies on reinforcing soils with BF will contribute to the literature.

- In these future studies, the clay type and properties will differ, and preparing a large number of mixtures with varying fiber ratios, lengths, and water contents will provide more data.
- As the input data increase in ELM analyses, the accuracy of the predictions will also improve, which is highly significant.
- A potential limitation of this study is the use of only one type of clay (kaolin) and BF with a length of 24 mm. Future research could explore the application of ELM to other soil types and examine the performance of reinforcement techniques using fibers of different types and lengths.
- Future work may also consider investigating the effect of environmental factors, such as temperature and humidity, on the compressive strength of fiber-reinforced soils to enhance the robustness of the model in real-world conditions.

This study is important as it demonstrates that the use of BF in soil reinforcement works can provide significant benefits due to its natural, sustainable, environmentally friendly characteristics and the abundance of its raw material.

## Figures and Tables

**Table 1:** Mechanical and physical properties of BF.

Feature	Value
Length of fiber (mm)	24
Diameter of monofilament Çapı (µm)	15 ± 1.5
Humidity, Max (%)	2
Modulus of elasticity (GPa)	90
Tensile strength (MPa)	3000
Thermal conductivity (W/mK)	0.031–0.038
Elongation at break (%)	3.5
Density (g/cm^3^)	2.63

**Table 2:** The results of experimental studies [24].

W Ratio (%)	Sample	K Ratio (%)	BF Ratio (%)	Average q_u_ (kPa)
20	K	100	0	102.40
	K + 1% BF	99	1	523.83
	K + 2% BF	98	2	685.31
	K + 3% BF	97	3	445.06
25	K	100	0	294.08
	K + 1% BF	99	1	807.40
	K + 2% BF	98	2	720.76
	K + 3% BF	97	3	705.00
30	K	100	0	96.07
	K + 1% BF	99	1	580.53
	K + 2% BF	98	2	630.23
	K + 3% BF	97	3	557.22
35	K	100	0	47.09
	K + 1% BF	99	1	51.89
	K + 2% BF	98	2	64.85
	K + 3% BF	97	3	47.95

W: water, K: kaolin clay, BF: basalt fiber.

**Table 3:** Comparison of the experimental results by the literature with the estimated results by ELM.

Sample	Inputs			Experimental Output	ELM Output
	Water Ratio	Clay Ratio	BF Ratio		
1	20	100	0	102.40	102.54
2	20	99	1	523.83	523.97
3	20	98	2	685.31	685.85
4	20	97	3	445.06	444.92
5	25	100	0	294.08	294.32
6	25	99	1	807.40	807.43
7	25	98	2	720.76	720.53
8	25	97	3	705.00	705.01
9	30	100	0	96.07	95.95
10	30	99	1	580.53	580.82
11	30	98	2	630.23	630.37
12	30	97	3	557.22	556.43
13	35	100	0	47.09	47.62
14	35	99	1	51.89	51.85
15	35	98	2	64.85	64.83
16	35	97	3	47.95	47.98

## Data Availability

The original contributions presented in this study are included in the article; further inquiries can be directed to the corresponding author.
